# A case report of postoperative poor drainage of perianal abscess in an obese diabetic patient treated with comprehensive therapy

**DOI:** 10.1093/jscr/rjag349

**Published:** 2026-05-06

**Authors:** Fang Li, Mian Qin, Zhen Lei, Ying Lu, Yuming Yuan, Xiaobin Shao, Rongqing Zhao

**Affiliations:** Department of Critical Care Medicine, Zhenhai Hospital of Traditional Chinese Medicine, No. 51 Huancheng West Road, Zhenhai District, Ningbo City, Zhejiang Province 315200, China; Department of Anorectal Surgery, Zhenhai Hospital of Traditional Chinese Medicine, No. 51 Huancheng West Road, Zhenhai District, Ningbo City, Zhejiang Province 315200, China; Department of Anorectal Surgery, Zhenhai Hospital of Traditional Chinese Medicine, No. 51 Huancheng West Road, Zhenhai District, Ningbo City, Zhejiang Province 315200, China; Department of Anorectal Surgery, Zhenhai Hospital of Traditional Chinese Medicine, No. 51 Huancheng West Road, Zhenhai District, Ningbo City, Zhejiang Province 315200, China; Department of Anorectal Surgery, Zhenhai Hospital of Traditional Chinese Medicine, No. 51 Huancheng West Road, Zhenhai District, Ningbo City, Zhejiang Province 315200, China; Department of Anorectal Surgery, Zhenhai Hospital of Traditional Chinese Medicine, No. 51 Huancheng West Road, Zhenhai District, Ningbo City, Zhejiang Province 315200, China; Department of Anorectal Surgery, Zhenhai Hospital of Traditional Chinese Medicine, No. 51 Huancheng West Road, Zhenhai District, Ningbo City, Zhejiang Province 315200, China

**Keywords:** perianal abscess, postoperative complications, obesity, diabetes mellitus, type 2, surgical drainage, multidisciplinary treatment

## Abstract

Postoperative inadequate drainage of perianal abscess is prevalent in obese patients with diabetes, caused by obesity-related high tissue pressure and diabetes-induced impaired immunity/microcirculation. We report a 25-year-old obese male (BMI 37.9 kg/m^2^) with newly diagnosed type 2 diabetes who developed recurrent abscess on postoperative Day 3 after surgery for perianal abscess, anal fistula, and mixed hemorrhoids. He was treated with active catheter drainage, ceftriaxone-based targeted anti-infection (guided by *Klebsiella pneumoniae* culture), and metformin + liraglutide for glucose control. The patient fully recovered with normalized inflammatory markers and blood glucose, without recurrence or anal fistula formation at 1-year follow-up. This validates the efficacy of the comprehensive strategy for high-risk patients with long-term clinical outcomes.

## Introduction

Postoperative poor drainage of perianal abscess hinders recovery, especially in obese patients (BMI ≥30 kg/m^2^) and those with diabetes [1]. Obesity causes premature drainage orifice closure via high tissue pressure, while diabetes impairs immune function, forming a vicious circle [1]. Traditional packing after incision and drainage is ineffective and painful [2], while antibiotics benefit high-risk patients [3]. This case explores the efficacy of a comprehensive strategy integrating active drainage, targeted anti-infection, and metabolic regulation, with extended follow-up to evaluate the long-term outcome of fistula formation, a key complication of perianal abscess.

## Case report

**Figure 1:**
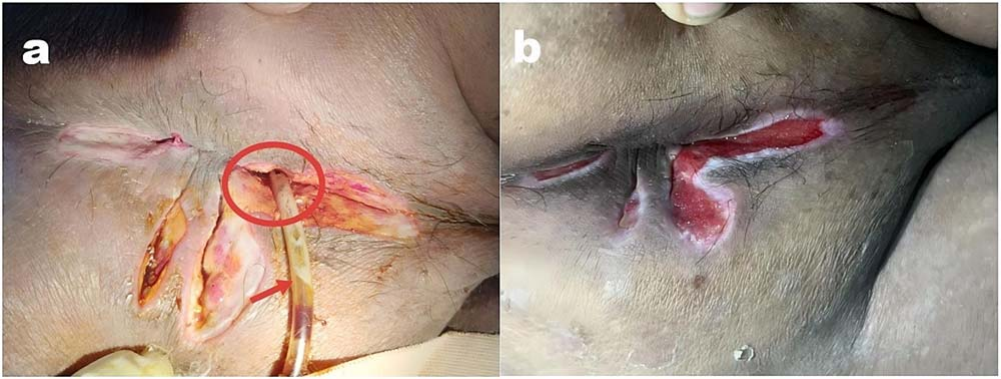
(a) Wound on postoperative Day 3: The circle indicates the recurrent abscess site in the anterior rectal space due to poor drainage, and the arrow points to an indwelling 14Fr rubber tube with pus drainage visible; (b) wound on postoperative Day 22: The wound is clean and essentially healed.

A 25-year-old male presented with 3-day perianal swelling and pain. He was severely obese (120 kg, 1.78 m, BMI 37.9 kg/m^2^) with preoperative random blood glucose 9.52 mmol/L and high-sensitivity C-reactive protein 56.12 mg/L. Emergency lumbar anesthesia was performed for incision and drainage, fistulectomy, and hemorrhoidectomy without packing [2]. On postoperative Day 3, incision closure with pus overflow confirmed recurrent abscess. Pus culture identified ceftriaxone-sensitive *Klebsiella pneumoniae*, and glycosylated hemoglobin 7.9% confirmed type 2 diabetes. Intervention included: (i) Active catheter drainage (14Fr rubber tube) with daily saline irrigation [4]; (ii) Ceftriaxone 2.0 g IV daily [3]; (iii) Metformin + liraglutide for glucose control [1, 4]. As shown in Fig. 1a, the indwelling drainage tube and recurrent abscess site were clearly displayed during the intervention period. Symptoms improved within 24 hours; the drainage tube was removed on Day 5. By postoperative Day 7, inflammatory markers and fasting blood glucose normalized. At postoperative Day 22, the wound was clean and essentially healed (Fig. 1b). No recurrence, anal fistula formation or other complications were noted at 1-year follow-up.

## Discussion

### Pathogenesis and clinical challenges

Postoperative poor drainage in this patient stemmed from synergistic mechanical and biological factors: obesity-induced high perianal tissue tension compressed drainage channels, while diabetes caused immune dysfunction and microcirculatory disorders, facilitating bacterial persistence [1, 4]. Perianal abscesses treated with incision and drainage have an ~8.82% recurrence rate and 45.58% anal fistula incidence [5]. This young male with extensive abscesses, obesity, and diabetes was extremely high-risk, requiring targeted solutions for ‘poor drainage’ and ‘metabolic abnormalities.’

### Rationale of comprehensive treatment

Active catheter drainage avoids the limitations of traditional packing (failure in obese patients, pain, delayed healing) [2]. Supported by the 2022 PPAC2 trial (non-packing reduced pain and improved healing) [2], it maintains drainage patency and facilitates irrigation, ideal for deep abscesses and obesity [6]. Precise anti-infection with ceftriaxone (guided by culture) reduces recurrence and fistula risk [3], critical for immunocompromised diabetic patients [6]. Systematic metabolic regulation with metformin + liraglutide addressed the synergistic risk of diabetes and obesity (OR = 2.098 when coexistent) [1], improving immunity and infection control [4].

### Clinical implications

For obese diabetic patients with perianal abscess, postoperative management should follow ‘proactive prevention → early detection → comprehensive intervention’: prioritize preoperative BMI and blood glucose evaluation, promptly detect poor drainage for early catheter drainage, use antibiotics based on etiological testing, and collaborate with endocrinology for metabolic control [1, 3, 4, 7]. Extended follow-up (at least 1 year) is recommended for this high-risk population to fully assess the occurrence of delayed complications such as anal fistula, which is a key part of clinical outcome evaluation. Transperineal POCUS (rapid, radiation-free) is valuable for dynamic drainage monitoring [8], with potential for optimizing preoperative evaluation and follow-up. Combined with the visual presentation in Fig. 1, clinicians can more intuitively assess the drainage status and wound healing process, improving the accuracy of postoperative management.

## Conclusion

The mechanism of postoperative poor drainage in obese diabetic patients involves synergistic mechanical obstruction and biological imbalance. The comprehensive strategy of ‘active catheter drainage + precise anti-infection + systematic metabolic regulation’ effectively improves the long-term clinical outcomes of high-risk patients, reduces recurrence and prevents anal fistula formation. It provides practical guidance for clinical practice and aligns with the trend of individualized, multidisciplinary and evidence-based treatment [6]. The changes in wound healing presented in Fig. 1 also visually confirm the effectiveness of the comprehensive treatment strategy proposed in this case.

## Data Availability

No datasets were generated or analyzed in this study.
